# Supplementary figures and images for: Modelling the factors affecting the probability for local rabies elimination by strategic control
Source: PLoS Negl Trop Dis. 2021 Mar 4;15(3):e0009236. doi: 10.1371/journal.pntd.0009236 (PMC7963038; doi:10.1371/journal.pntd.0009236)

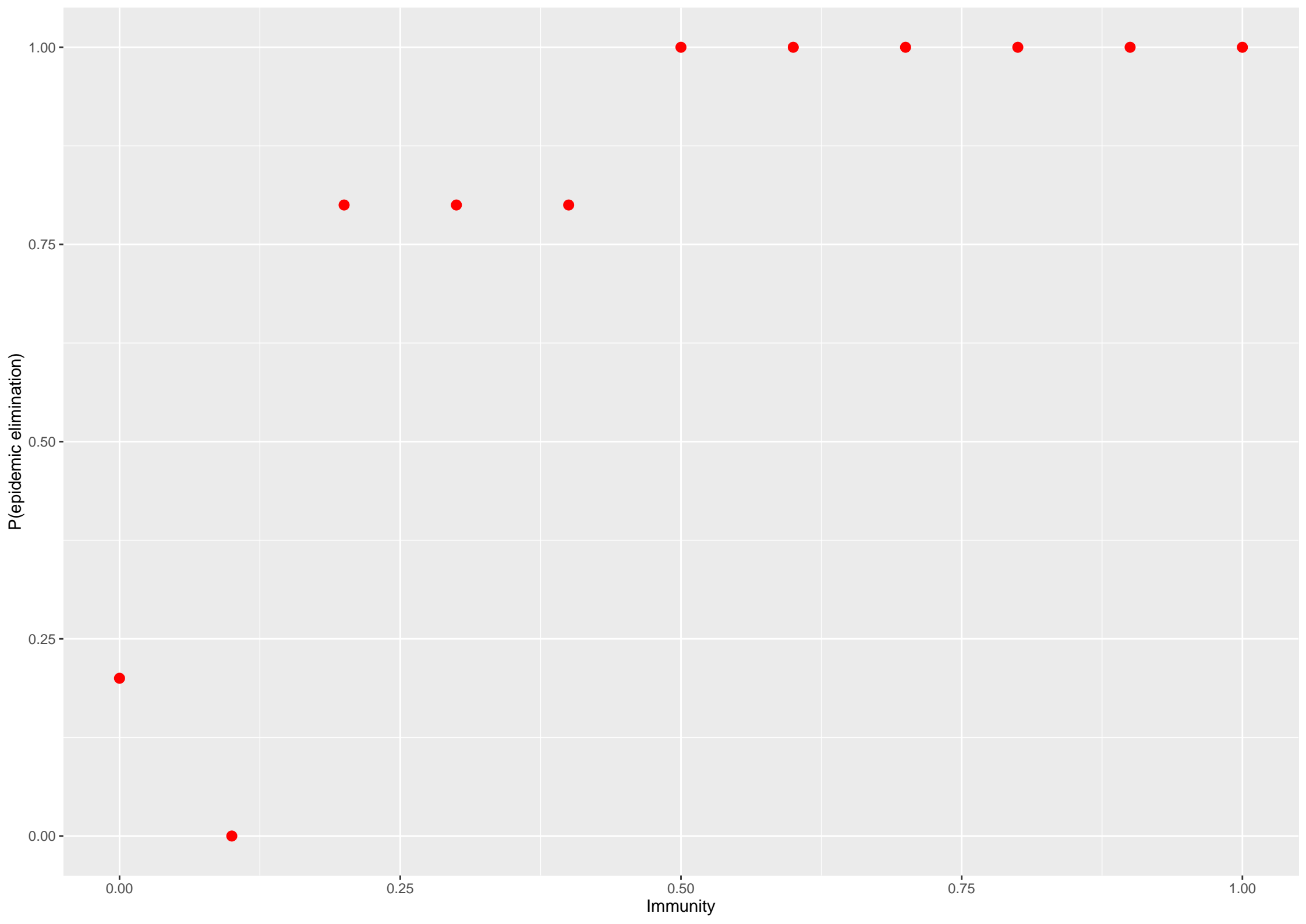

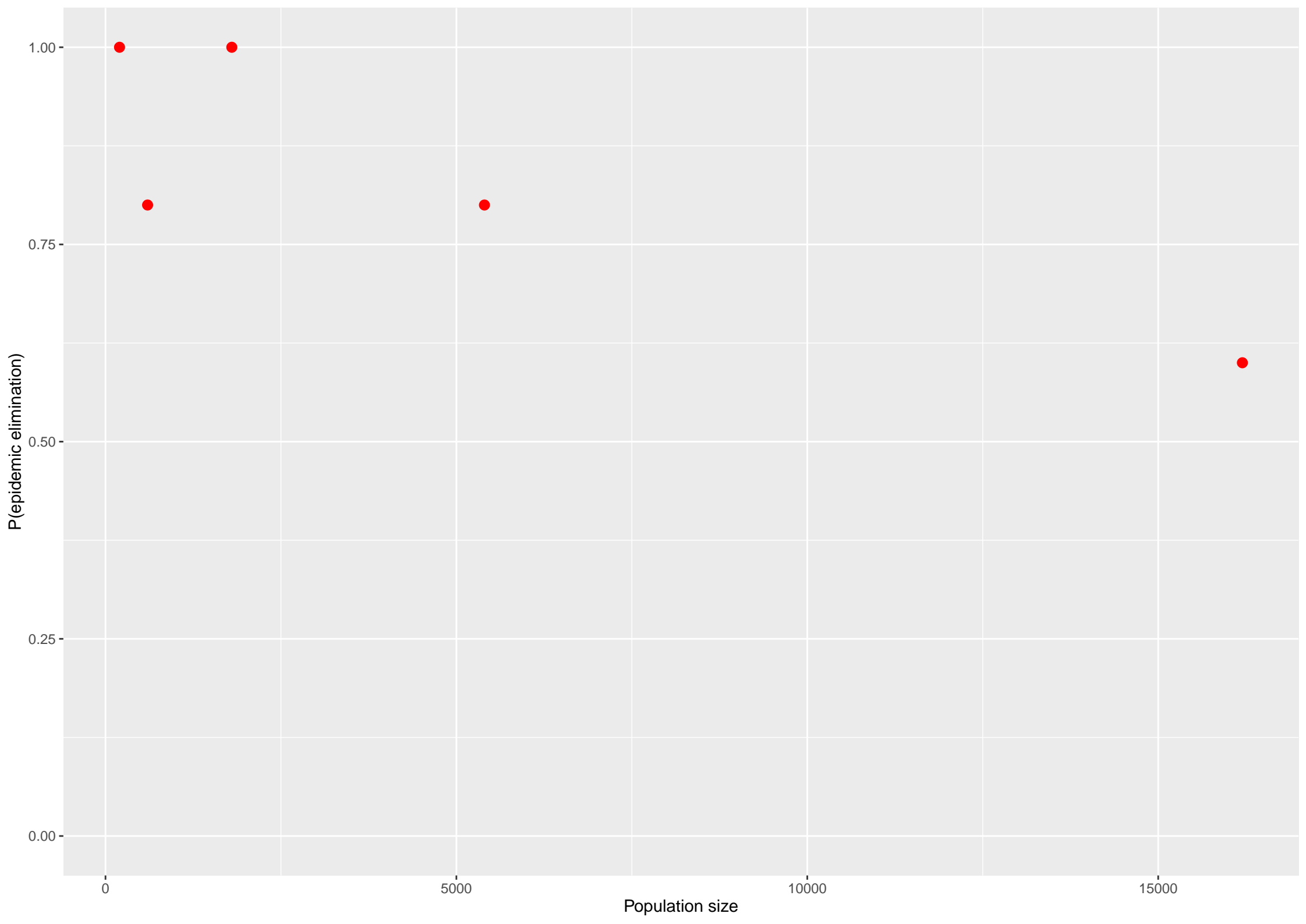

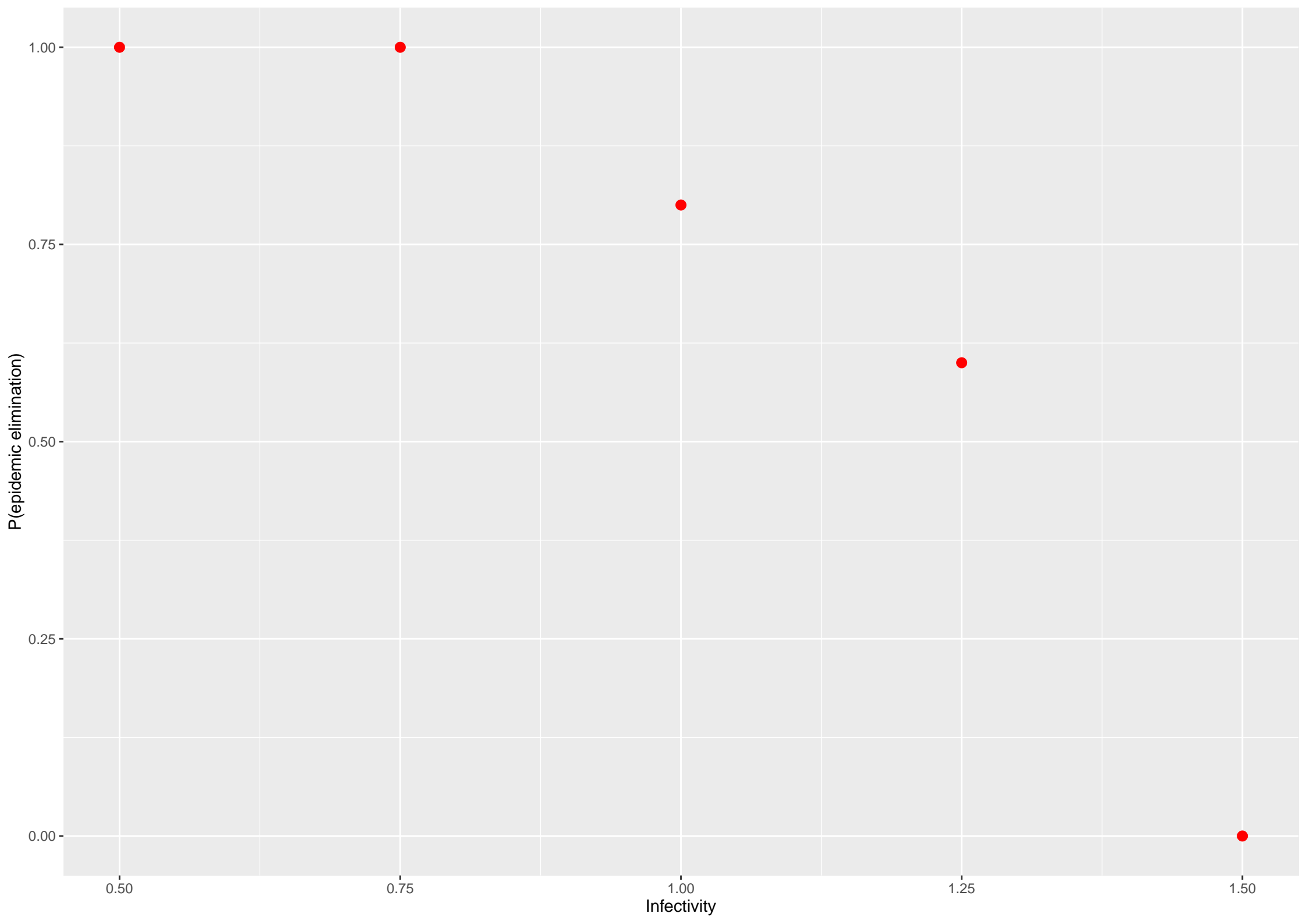

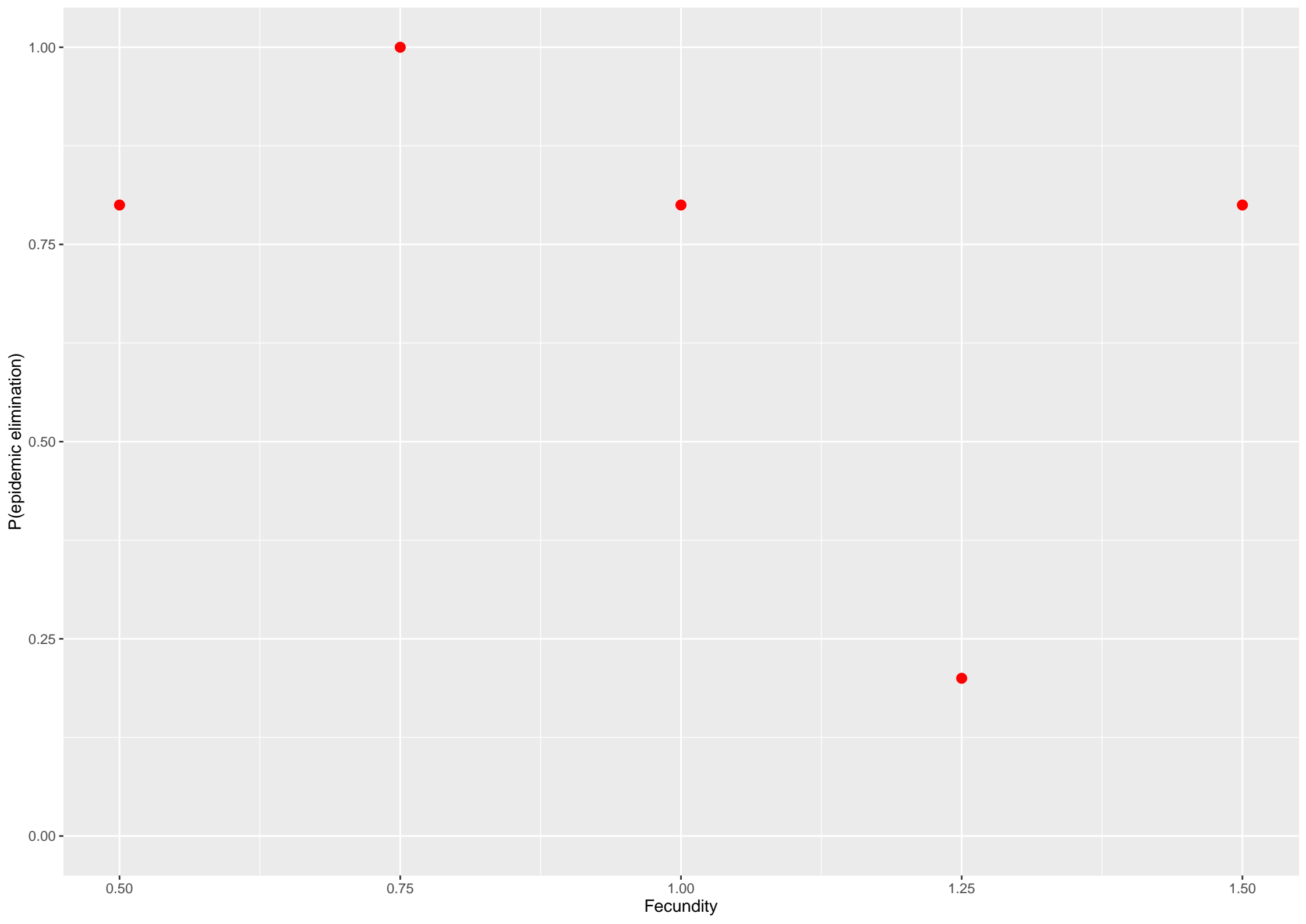

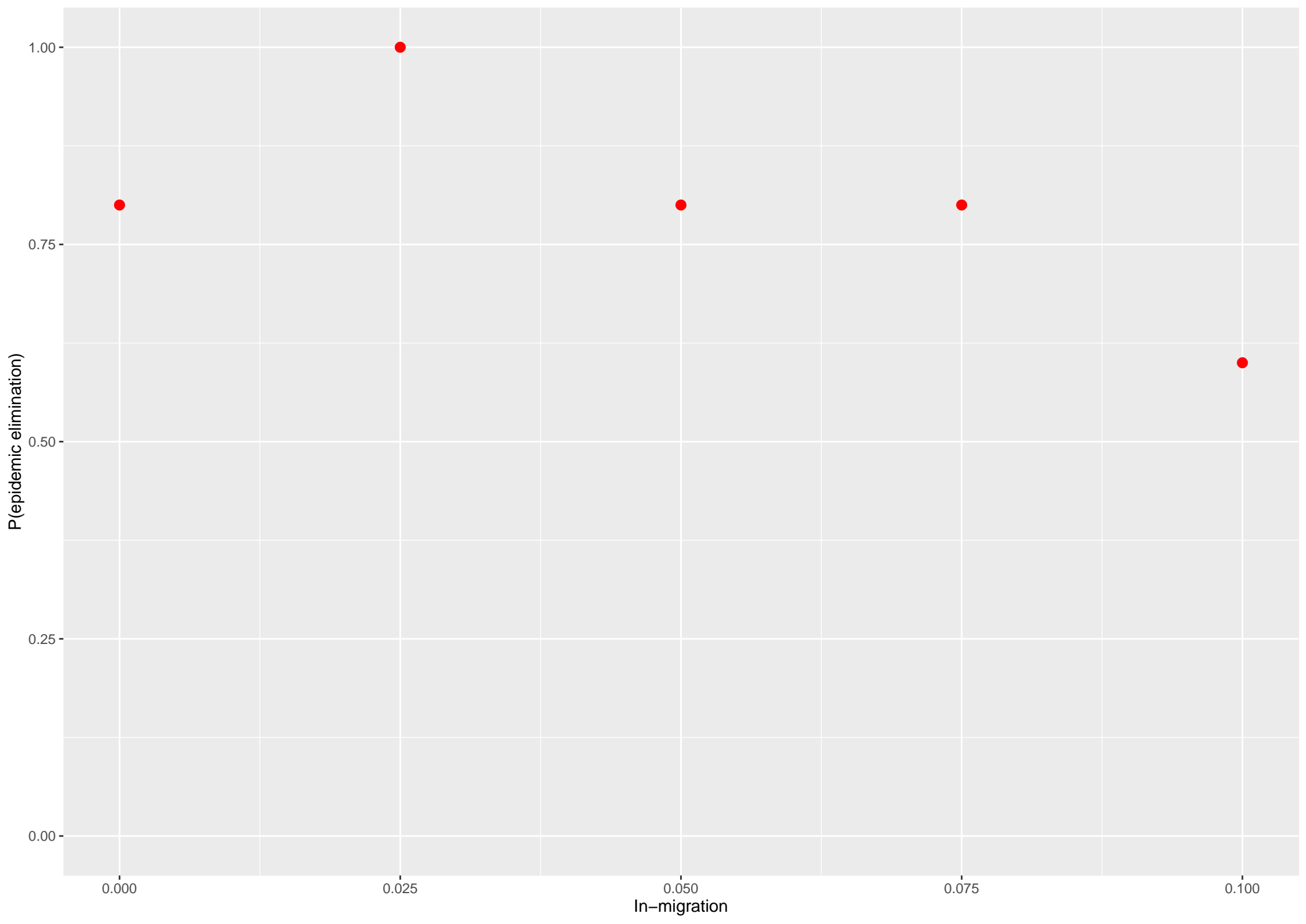

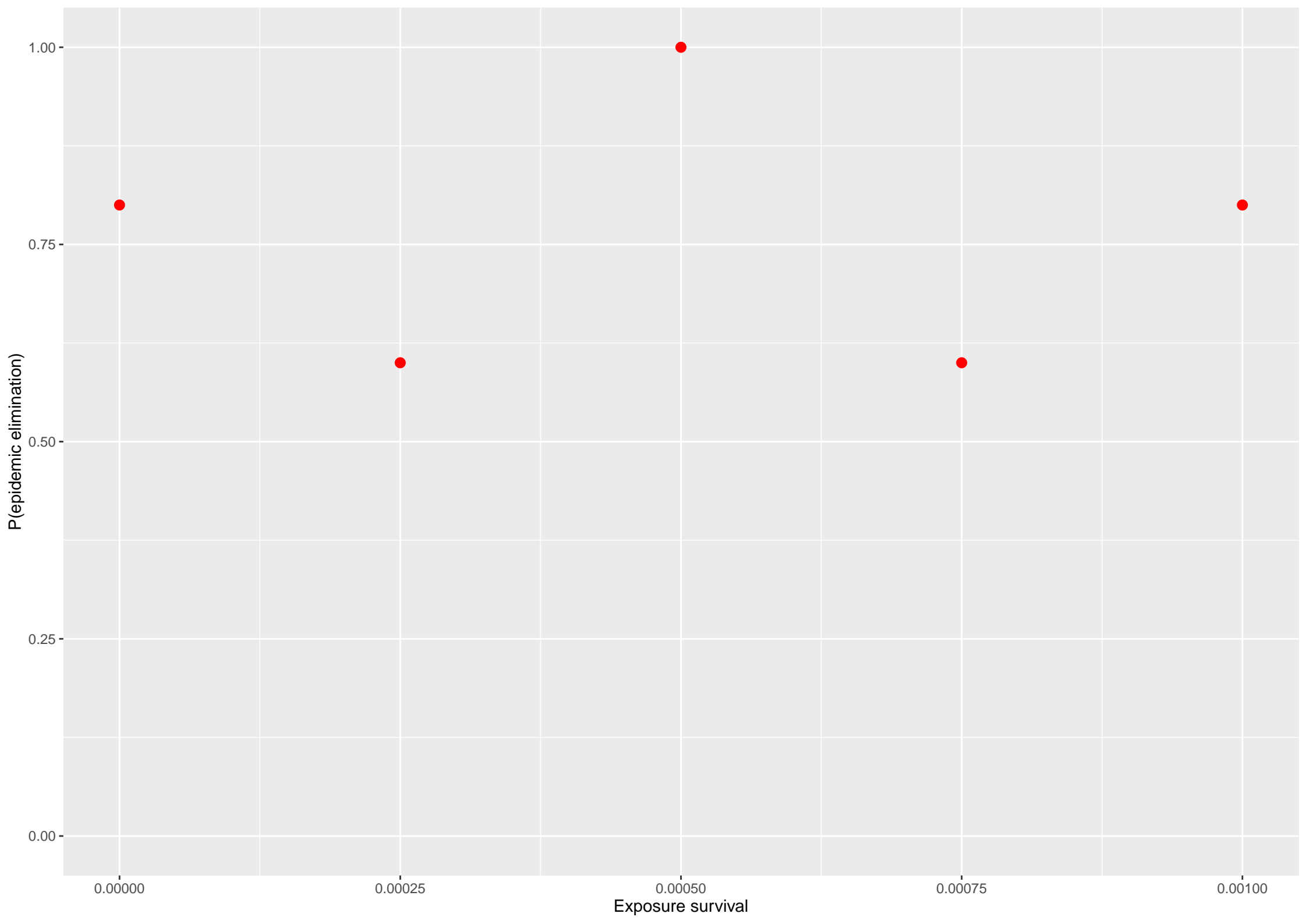

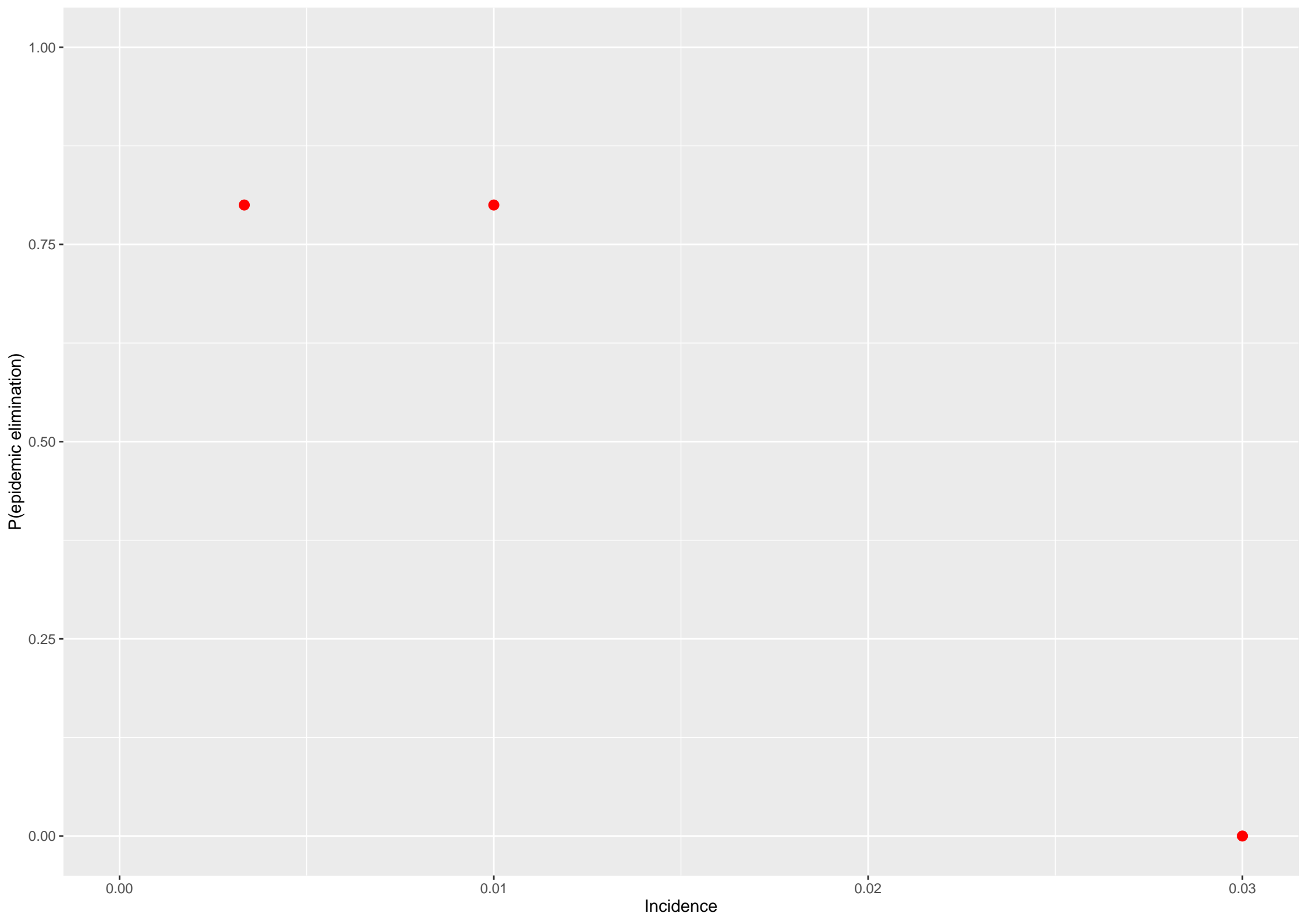

Supplement: S1 Fig — The model parameters plotted against the probability for elimination. (PDF) [file pntd.0009236.s002.pdf]
