# Supplementary material for: Modelling the factors affecting the probability for local rabies elimination by strategic control
Source: PLoS Negl Trop Dis. 2021 Mar 4;15(3):e0009236. doi: 10.1371/journal.pntd.0009236 (PMC7963038; doi:10.1371/journal.pntd.0009236)
Supplement: Attachment — Submitted filename: Response letter.docx [file pntd.0009236.s010.docx]

**To:** the Editorial Review, PLOS NTD

**Re:** Significant revisions to the submission to PLOS NTD of a paper titled “*Modelling the factors affecting the probability for local rabies elimination by strategic control”*

The reviewers are thanked for their thought-provoking comments that have led (in our view) to a much-improved paper of better quality and better utility.

All changes are highlighted in green in the ‘highlight’ version.

**Major changes:**

1. **Modelled the effect of disease re-introductions by adding an initial incidence variable**

Re-introductions represent the start of an epidemic with minimal incidence initially (before the epidemic gathers momentum). In mature epidemics with no interventions, maximal incidence can be expected. By varying the incidence between a minimum and a maximum we could simulate (re-)introductions compared to endemic situation.

We did not specifically include a stochastic variable with a probability for a re-introduction. The reasons why have opted not to do are outlined below.

a. Because the probability for a re-introduction over a one-year period is relatively low, it would require substantially more iterations of every unique parameter-combination to be able to draw useful conclusions.
b. The effect of frequent re-introductions would obscure the true nature of the effect of the other modelled parameter-combination.
c. A typical example would be when a chance re-introduction occurs shortly before the end of the one-year simulation.

We therefore reckoned that distinguishing between the risk to a dog population from re-introduction versus endemic situations offers a preferable alternative. (This was done by adding the initial incidence variable.)

This is the biggest change to the paper as it required a change to the foundation of the study design. It is therefore also reflected in all the sections of the paper.

2. **Intermediate models**

We better described the specifications of all the intermediate models. We reported the residual deviance and how it changed in the search for the optimal model.

| Methods, The regression models | Lines: 207-229 (Including Table 2) |
| --- | --- |
| Supporting Information | S5 Script (Model specifications) |
| Supporting Information | S6 Text (Model results) |

3. **Improved Supporting information**

We have included all scripts and data to reproduce the paper in total. (Because the complete analyses can take several weeks to compute we, have also made intermediate data results available at https://doi.org/10.5281/zenodo.4362497)

4. **Appropriate placement of methods, results and discussion.**

We have re-arranged the paper to better separate methods, results and discussions.

5. **Improved that paper’s ability to meet the objective of assisting campaign managers with practical application examples.**

We have included more suggestions for practical application in the discussion section.

6. **Major assumption of sub-population**

We have emphasized in the limitations section the major assumption of sub-populations.

7. We have **clarified the scope** of the paper by adding ‘dog’ or ‘dog-mediated’ in several places. (See minor changes.)
8. **New section added to introduction (lines 83-95).**

This paragraph explains the reason why models are suitable to address the identified need and briefly introduces bio-economic, stochastic transmission models.

9. ddd

**Minor changes**

| **Description** | **Lines (1^st^)** | **Lines (new)** |
| --- | --- | --- |
| **Abstract** | | |
| ‘rife’ changed to ‘widespread’ | 17 | 17 |
| ‘human’ added to give ‘dog-mediated human rabies’ | 20 | 20 |
| ‘onus’ changed to ‘responsibility’ | 20 | 21 |
| ‘success’ changed to ‘successful elimination’ | 22 | 23 |
| ‘well known’ changed to ‘often quoted’ | 23 | 23 |
| Factors named specifically | 29 | 26-28 |
| Added ‘and lower incidence’ | 32 | 32 |
| Included ‘dog-mediated’ to be clear that the paper is not about wildlife rabies. | 34 | 36 |
| **Author summary** | | |
| Added ‘dogs’ | 37 | 40 |
| Changed order: we moved the statement on infectivity before the statement on population size. | 43 | 43 |
| Included a statement of donors. |  | 50-51 |
| **Introduction** | | |
| ‘transmit’ changed to ‘are responsible for’ | 52 | 53 |
| Removed ‘scratches’ as a source of rabies infection. Although true its inclusion adds nothing to this paper. | 53 | 54 |
| Changed the wording of the statement on the relative rarity of rabies to be clear that it does not refer to the global prevalence but rather the incidence rate. | 55 | 57-61 |
| Clarified a statement on the barriers to elimination not be misread as saying that quality vaccines are not important. | 58-59 | 56-64 |
| ‘through death’ changed to ‘rapid population turnover rates’ | 63 | 68 |
| Added a third reference to make the case from the literature stronger that dog ecologies differ between settings. | 66 | 71 |
| Added ‘vaccination’ in front of ‘campaigns’. | 68 | 73 |
| ‘reported incidence’ changed to ‘reported cases’ | 71 | 76 |
| ‘spatially separated’ added in front of ‘sub-populations’ | 71 | 77 |
| **Methods** | | |
| ‘Six’ changed to ‘seven’ | 81 | 98 |
| Added ‘dog’ in front of ‘rabies | 82 | 99 |
| Changes in the size of the dataset were edited | 86, 88-89 | 103, 105, 107 |
| The format of reporting numbers in the thousands was changed from 999 999 to 999,999. | All through | All through |
| Success was better defined as representing an elimination event. | 88 | 105-106 |
| Table 1: the parameter space was better described for each variable. | 91 | 109 |
| Sentence added to the Methods summary to describe calculations later in the text. |  | 118-120 |
| **Methods, The disease transmission model** | | |
| ‘peer-reviewed’ added | 103 | 123 |
| Moved sentence to make flow more logical | 105-108 | 124-126 |
| Added description of new variable: ‘incidence’ |  | 131-135 |
| Added a fourth reference to make the case from the literature stronger that density dependent transmission of dog rabies is not widely accepted. | 115 | 139 |
| Expanded on the explanation why it would not be a good idea to include dog density in the model. | 117-119 | 140-147 |
| A new paragraph was devoted to better discuss fecundity. | 122-125 | 148-153 |
| We re-wrote the paragraph on exposure survival to be more logical. We better explained the choice for the limits of the parameter space. | 126-135 | 157-165 |
| Added another recent reference (from 2020) to support the idea that rabies survival requires meta-populations. | 137 | 167 |
| Added a reference to support mortality related extinctions of small dog populations. | 142 | 172 |
| Added a paragraph to explain how the new incidence variable works. |  | 178-185 |
| Added a justification to end the simulation after one year. | 150 | 191-194 |
| Adapted numbers for extinction rates due to new data set. We also pointed out that extinction events only occurred at zero herd immunity. | 155-156 | 199-201 |
| **Methods, The regression models** | | |
| Included ‘(covariate pattern)’ for the benefit of some readers. | 159 | 207 |
| Named the intermediate models to report on their performance. |  | 211, 213, 215, 217 |
| We included a better explanation why we sought the optimal model the way we did. |  | 217-220 |
| We changed the wording why we chose the AIC as the main criteria to seek the optimal model. | 168-169 | 222-223 |
| Table 2 and S6 Text were added to report on the performance of the intermediate models. |  | 226-228 |
| Report on the optimal model moved to Results section | 174-181 | 334-341 |
| Figures 6 and 12 added for the incidence variable |  | 250-252; 283-287 |
| All plots had to be redone. | 187-244 | 235-297 |
| Explanation added in the Methods section to improve the flow of the paper and to improve the explanation of the logic behind the N’Djamena example. | 342-351 | 302-310 |
| Explanation added in the Methods section to improve the flow of the paper and to improve the explanation behind the logic of the sterilizations vs. vaccinations example. | 367-388 | 312-330 |
| **Results** |  |  |
| The number of terms in the optimal model changed from 76 to 70 (not by intention). | 174 | 334 |
| ‘Initial population size’ was re-written to reflect new numbers. | 248-256 | 344-351 |
| ‘Infectivity’ was re-written to reflect new numbers. | 258-269 | 353-364 |
| ‘Fecundity’ and ‘In-migration’ results were split. | 271-282 | 366-378 |
| ‘Exposure survival’ results were combined with that of ‘In-migration’ | 284-289 | 374-378 |
| Interpretations were removed from the results section. | 281-282 |  |
| Paragraphs added for the results of the two worked examples. |  | 400-416 |
| **Discussion** | | |
| The currency of cost calculations of sterilizations versus vaccinations was changed to USD. (Previously in Discussion section.) | 387 | 415 |
| Possible reasons why small sub-populations lead to increased elimination events was added. |  | 419-429 |
| A paragraph was added addressing the concern of human-mediated, long distance movements of dogs. (This is further addressed under limitations.) |  | 450-454 |
| A brief explanation of the meaning of betweenness centrality was added. | 326 | 457-458 |
| The implications of the N’Djamena results are discussed. |  | 468-481 |
| The importance of collaborative efforts to address rubbish dumps was added. |  | 485-486 |
| Implications for campaign managers on the fecundity results was added. |  | 494-501 |
| Implications for campaign managers on the in-migrations results was added. |  | 506-510 |
| A paragraph was added discussing the implications of the results of the new variable ‘incidence’. |  | 519-533 |
| ‘dog’ was added in front of ‘rabies’ | 407 | 537 |
| ‘…is in itself an awareness campaign…” was changed to “…in itself can be an awareness camapgin…” | 409 | 539-540 |
| We have removed a controversial reference to the effect incomplete immune-responses that lead to paralytic rabies. | 412 | 539 |
| **Limitations** | | |
| The section is a complete re-write. It now addresses 2 new major limitations:   1. The assumption of *sub-populations.* 2. Running the simulation for 1 year only. |  | 543-555 |
| The limitation on the source of the parameter values was re-written to be clearer. | 418-423 | 556-563 |
| **Conclusions** | | |
| We better emphasized that vaccinations resulting in 70% herd immunity is the surest route to elimination. | 433 | 572-574 |
| The three most important factors to effect elimination (besides vaccination) have been listed. |  | 577 |
| A closing sentence emphasizing the importance of understanding dog ecology has been added. |  | 577-580 |
| **Supporting information** | | |
| Completely re-done. The supporting information is complete enough to reproduce the paper. Intermediate results (large files) are also available at <https://doi.org/10.5281/zenodo.4362497> for readers wishing to reproduce only some of the intermediate sections. | 538-539 | 697-705 |

**What we have not changed and our reasons why not:**

1. Density dependent transmission
   a. The effect of density dependent transmission is higher bite rates per rabid dog. The infectivity variable is exactly this.
      We are not aware of literature that can inform a quantitative relationship between dog population density and bite rates. Instead, there is literature to support that there is no such relationship.
   b. As authors we have also struggled with the intuitive deduction that higher dog densities would have higher bite rates. As mentioned in the paper, this remains controversial, but we felt that currently the strongest literature favours a frequency dependent approach rather than a density dependent approach. It could be that dog social behaviour may dictate that at higher dog densities home range sizes are proportionally smaller. The effect would be equal contact rates at higher densities compared to lower densities. (As this is mere speculation, we have not added this possible explanation to the paper.)
   c. If density dependent transmission is true, higher dog densities would have higher bite rates. This has been modelled through the infectivity variable.
2. We have not included rates of challenge survival above the originally submitted maximum of 0.1%.
   a. Our aim with the paper is to parameterize as good as we can using the available scientific knowledge. Whereas serological surveys of dogs in rural communities have sometimes found positive titres in apparent unvaccinated dogs in excess of 0.1%, we do not view these studies as trustworthy methodology. (We have ourselves seen school kids bringing local dogs for vaccination that they or their families do not own.) We have therefore focused on the only controlled experiment we know of.

We have tried to better explain our reasoning behind this decision (lines 157-165).

We hope you share our enthusiasm for this work and would agree to publish it in PLOS NTD.

Yours sincerely,

Dr Johann Kotzé
