# Supplementary material for: Modelling the factors affecting the probability for local rabies elimination by strategic control
Source: PLoS Negl Trop Dis. 2021 Mar 4;15(3):e0009236. doi: 10.1371/journal.pntd.0009236 (PMC7963038; doi:10.1371/journal.pntd.0009236)
Supplement: Attachment — Submitted filename: Response letter.docx [file pntd.0009236.s011.docx]

**To:** the Editorial Review, PLOS NTD

**Re:** Significant revisions to the submission to PLOS NTD of a paper titled “*Modelling the factors affecting the probability for local rabies elimination by strategic control”*

The reviewers are thanked for recognising the improvements made in this version. Their further comments are also seen as very valuable, especially where possible misunderstandings have been highlighted.

Line references are for the ‘Track Changes’ version only.

**Major comments:**

1. **Campaigns that run beyond 1-year**

There are two separate issues that were identified by the reviewers. The first had to do with potential confusion leading readers into thinking that campaign managers should only attempt to eliminate rabies within one year. This is a very dangerous and important misconception to be clear about. We decided to completely remove the sentence that gave rise to the confusion. (Lines 194-196). We better conveyed the intended meaning in both the Limiations and Discussion sections (lines 546-556 and lines 567-578).

The second issue identified by the reviewers deals with the limitation of the model. Reviewers rightly mention that typical campaigns run over several years which creates a need for longer projections from the model. We attempted to address this very important concern extensively. We firstly recognise the problem as identified by the reviewers (lines 568-569) then we explain why distant projections are dangerous when stochastic models are used (lines 569-573). Finally, we provide a solution to the limitation that we believe would be more accurate and precise than modelling over several years (lines 573-575).

We have also included a paragraph in the Discussion section to advise campaign managers how to account for waning herd immunity (lines 546-556). We feel that the advice we provide also addresses a scenario where campaigns are repeated annually for several years.

2. **Procedure and capture costs of the vaccination versus sterilization example**

Reviewers appear not to have been able to view the full extent of the data contained in Table 3. Unfortunately, the PLOS submission guidelines dictate that table margins should not be adjusted to fit the submitted manuscript. This is understandable from an editing point of view. Subsequently, when the manuscript is converted to pdf for reviewers, the full content of the table is not visible (at least, this is what one of the reviewers concluded has happened.)

For the benefit of the reviewers, we post an size-adjusted, verbatim copy of Table 3 below:

|  | **Sterilization** | **Vaccination** |
| --- | --- | --- |
| Baseline population before intervention is **16,200 dogs** that is **30% immune** irrespective of demographics. | | |
| 61% are adult dogs of which 38% are female. **Fecundity factor is 1**. | | |
| Number of dogs eligible for procedure: | 16,200 x 0.61 x 0.38 = 3,755 | 16,200 |
| Number of dogs already vaccinated/sterilized: | 0 | 30%; 16,200 x 0.3 = 4,860 |
| Number of dogs to receive procedure: | 939 (0 - 25%) | 11,340 (30 - 100%) |
| Capture costs are $0.58; $0.99; $1.13; $2.22 per dog in the population brackets 0 - 25%; 26 - 50%; 51 -75%; 76 - 100% | | |
| Costs to capture dogs for interventions: | $544.62 | $15,913.98 |
|  | =$0.58 x 939 | =($0.58 x 0) + ($0.99 x 3235) + ( $1.13 x 4050) + ($2.22 + 4050) |
| Procedural costs are $19.80 per bitch sterilization and $0.16 per dog vaccination. | | |
| Total procedural cost for all dogs: | $18,592.20 | $1,814.40 |
|  | =$19.80 x 939 | =$0.16 x 11340 |
| Total intervention cost: | $19,136.82 | $18,589.10 |
| Demographic effect | Fecundity moves from 1 to 0.75. | Herd immunity moves from 0.3 to 1. |
| P(elim.) before intervention: | 0.37 | 0.37 |
| P(elim.) after intervention: | 0.40 | 1.00 |
| USD^a^ spent per percentage point gained in P(elim.): | $6,378.94 | $295.07 |
|  | |  |

We hope that when the reviewers can view the full table the answers to their concerns become evident. We opted not to change any of text in the methodology nor the table.

When only vaccinating, the total capture costs are high because capturing the last dogs to achieve high coverage come with higher capture costs. When only sterilizing the capture-costs are low because the number of dogs captured are much less. Of course, the procedure to sterilize is much more expensive than the procedure to vaccinate. Again, we feel that these points were already clear in the text and the table and we hope the reviewers will be satisfied.

**Minor changes**

| **Description** | **Lines** | |
| --- | --- | --- |
| **Introduction** | | |
| ‘follows’ changed to ‘approaches’ | | 90 |
| **Methods** | | |
| A reviewer said that: *“…to claim that the latter [density-dependent transmission] was suggested as an alternative for frequency-dependent transmission is historically not correct. Not long ago, rabies was considered an exclusively density-dependent transmitted disease”.*  To respond properly to the reviewer, we wanted to review the historic trend in the use of density-dependent versus frequency dependent canine rabies models. Luckily a review of canine rabies models by Rajeev et al. from 2019 summarized 51 models including a note on how transmission was modelled. Please find below a graph based on their data:  The raw data used to produce this is available here:  <https://github.com/mrajeev08/ModelingChapter/blob/master/data/modeling_studies.csv>  It is clear from the data that there is no scientific consensus on the subject yet. We believe the way we rephrased the sentence now better reflects the historic data.  We’ve also added a reference to the Rajeev et al. study. | | 138-141 |
| Added “…with time” to “…natural decline in immunity…”. | | 194 |
| **Discussion** | | |
| ‘follows’ changed to ‘approaches’ | | 431 |
| Added a reference suggested by a reviewer. | | 533 |
| Added “the”. | | 538 |
| Additional paragraph added (see Major Changes above). | | 546-556 |
| **Limitations** | | |
| Major edits to paragraph (see Major Changes above). | | 546-556 |

Density- vs Frequency-dependent canine rabies models

|  | Density | Frequency | NA |
| --- | --- | --- | --- |
| 1995 | 1 | 0 | 0 |
| 1996 | 0 | 0 | 1 |
| 1997 | 0 | 0 | 0 |
| 1998 | 0 | 0 | 0 |
| 1999 | 0 | 0 | 0 |
| 2000 | 0 | 0 | 0 |
| 2001 | 0 | 0 | 0 |
| 2002 | 1 | 0 | 1 |
| 2003 | 0 | 0 | 0 |
| 2004 | 0 | 0 | 0 |
| 2005 | 0 | 0 | 0 |
| 2006 | 0 | 0 | 0 |
| 2007 | 1 | 0 | 0 |
| 2008 | 1 | 0 | 1 |
| 2009 | 1 | 1 | 0 |
| 2010 | 2 | 0 | 1 |
| 2011 | 2 | 0 | 0 |
| 2012 | 4 | 0 | 1 |
| 2013 | 0 | 2 | 0 |
| 2014 | 1 | 0 | 1 |
| 2015 | 2 | 2 | 0 |
| 2016 | 3 | 4 | 0 |
| 2017 | 4 | 1 | 1 |
| 2018 | 3 | 2 | 2 |
| 2019 | 1 | 2 | 1 |

**Reviewer comments that we address without any changes to the manuscript:**

1. “*The low incidence of rabies is not due to a low basic reproductive number (COVID has a low R_0_ but high incidence)”.*

Diseases with shorter incubation periods will have higher incidence compared to disease long incubation periods and the same R_0._ This is because incidence is a rate that is measured over time. R_0_ is not a rate (at least not in terms of time). Therefore, diseases with long incubation periods will have low recorded incidence when R_0_ is held constant. Rabies has very long incubation periods compared to COVID.

2. *“The low incidence of rabies in endemic countries is because not all dogs infected (bitten) will develop rabies and in almost all endemic countries a certain percentage of the dog population is vaccinated”*

We have discussed in the paper the somewhat controversial issue of dogs immune-converting from natural exposure (without developing disease). This is probably not what the reviewer is referring to. If it is, we stand by our position expressed in the paper that the current evidence for such a phenomenon is not strong enough to conclude that it happens on a large scale.

If the reviewer meant that bitten dogs simply do not develop disease. Then we wholeheartedly agree. We distinguish a few times in the paper the difference between ‘bites’ and ‘effective bites’ (notably Table 1). R_0_ is calculated from incidence which implies ‘effective bites’ and not ‘bites’. R_0_ is also calculated assuming zero immunity in a population – although we have to agree that this assumption is often conveniently ignored.

3. The fact that no changes were made to Table 3 has already been discussed above.

We hope that the paper will now satisfy the reviewers in this form.

Yours sincerely,

Dr Johann Kotzé
